# Ets-1 deficiency alleviates nonalcoholic steatohepatitis via weakening TGF-β1 signaling-mediated hepatocyte apoptosis

**DOI:** 10.1038/s41419-019-1672-4

**Published:** 2019-06-12

**Authors:** Dechen Liu, Kai Wang, Kai Li, Rufeng Xu, Xiaoai Chang, Yunxia Zhu, Peng Sun, Xiao Han

**Affiliations:** 1 Key Laboratory of Human Functional Genomics of Jiangsu Province, Nanjing Medical University, Nanjing, China; 2 Department of Clinical Science and Research, Zhongda Hospital, School of Medicine, Southeast University, Nanjing, China

**Keywords:** Apoptosis, Mechanisms of disease, Non-alcoholic steatohepatitis

## Abstract

Hepatocyte apoptosis is a hallmark of nonalcoholic steatohepatitis (NASH) and contributes to liver injury, fibrosis, and inflammation. However, the molecular mechanisms underlying excessive hepatocyte apoptosis in NASH remain largely unknown. This study aimed to explore whether and how the v-ets avian erythroblastosis virus E26 oncogene homolog 1 (Ets-1) is involved in diet-induced hepatocyte apoptosis in mice. The study found that the expression level of hepatic Ets-1 was elevated in a NASH mouse model as a result of the activation of transforming growth factor beta1 (TGF-β1) signaling. In the presence of TGF-β1, phosphorylated mothers against decapentaplegic homolog 2/3 (p-Smad2/3) translocated to the binding sites of the *Ets-1* promoter to upregulate the expression of Ets-1 in primary hepatocytes. In addition, Ets-1 bound directly to phosphorylated Smad3 (p-Smad3), thereby preventing the ubiquitination and proteasomal degradation of p-Smad3 and enhancing the activity of TGF-β1/Smad3 signaling. Consequently, elevated Ets-1 stimulated TGF-β1-induced hepatocyte apoptosis. However, Ets-1 knockdown alleviated diet-induced hepatocyte apoptosis and NASH with reduced liver injury, inflammation, and fibrosis. Taken together, Ets-1 had an adverse impact on hepatocyte survival under TGF-β1 treatment and accelerated the development of NASH in mice.

## Introduction

Nonalcoholic fatty liver disease (NAFLD) is both common and chronic and occurs in ~25% of the general population in the world^1^. NAFLD includes a wide spectrum of disorders ranging from simple steatosis to nonalcoholic steatohepatitis (NASH). Approximately 30% of patients with NAFLD have NASH^2^. NASH is characterized by liver injury, inflammation, fibrosis and a high risk of cirrhosis and eventual hepatocellular carcinoma^3^. Although multiple pathogenic factors, including lipotoxicity, oxidative stress, and mitochondrial dysfunction, drive the transition from simple steatosis to NASH, the cellular and molecular occurrences that lead to NASH progression remain largely unknown^4,5^.

Increased apoptosis in hepatocytes is a crucial feature of NASH as demonstrated in both clinical and animal studies^6,7^. During the development of NASH, hepatocellular apoptosis activates the aberration of the liver’s regenerative responses, including fibrosis and inflammation^8,9^. Meanwhile, chronic inflammation in liver tissues stimulates macrophages to produce cytokines, which further promote hepatocyte apoptosis, thereby aggravating NASH^10^. Among the cytokines, transforming growth factor beta1 (TGF-β1) is closely associated with fibrosis and apoptosis, which plays a major role in the progression of NASH^11^. Besides activating hepatic star cells (HSCs) for extracellular matrix deposition^12^, TGF-β1 signaling was also over-activated in hepatocytes of patients with NASH^13^. It has been proven that the hepatocyte specific knockdown of TGF-β type II receptor (TβRII) preserves hepatocyte survival and protects against diet-induced NASH in mice^13^. Ramjaum et al. reported that hepatocyte apoptosis induced by TGF-β1 signaling was partially dependent on mothers against decapentaplegic homolog 3 (Smad3), which modulated the expression of certain apoptosis-associated proteins, such as BCL-2-interacting mediator of cell death (Bim)^14,15^. Thus, targeting TGF-β1/Smad3 signaling in hepatocytes is beneficial for alleviating apoptosis and NASH.

In this study, the dataset of patients with NASH from the Gene Expression Omnibus (GEO) database was analyzed, revealing a positive relationship between the expressions of transforming growth factor beta 1 (TGF-β1) and v-ets avian erythroblastosis virus E26 oncogene homolog 1 (Ets-1). Ets-1 belongs to the E26 transformation-specific sequence (ETS) transcription factor family and can combine with Smad3 and contribute to various regenerative responses, including immune cell differentiation, fibrosis and angiogenesis^16–18^. In addition, Ets-1 is closely associated with apoptosis, and our previous study demonstrated that Ets-1 protected β-cells against apoptosis^19–21^. Despite this, it is unclear as to whether Ets-1 is involved in TGF-β1/Smad3-induced hepatocyte apoptosis during the transition from steatosis to NASH. To test this hypothesis, the effect of Ets-1 on primary hepatocytes and mice fed an methionine choline-deficient (MCD) diet-fed was evaluated.

## Results

### The expression of Ets-1 was elevated in the liver tissues of mice with NASH

**Fig. 1:**
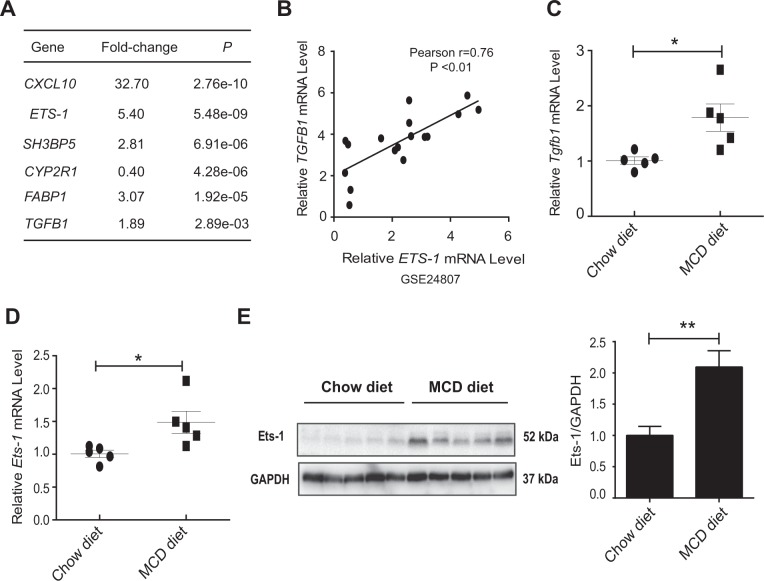
The expression of Ets-1 was associated with the progression of NASH. **a**, **b** Expression of *Ets-1* was examined in biopsies from normal controls (*n* = 5) and patients with NASH (*n* = 12) (GSE24807). **a** Some genes that changed significantly are listed. *CXCL10*, CXC motif chemokine ligand 10; *SH3BP5*, SH3 domain–binding protein 5; *CYP2R1*, cytochrome P450 2R1; *FABP1*, fatty acid-binding protein 1; *TGFB1*, transforming growth factor beta1. **b** The correlation between *ETS-1* and *TGFB1* was determined by using the linear regression test (*P* < 0.01). **c**–**e** WT mice were fed with chow diet (*n* = 5) and MCD diet (*n* = 5) for 8 weeks and sacrificed. Hepatic mRNA levels of *Tgfb1* (**c**) and *Ets-1* (**d**) were examined using qRT-PCR. **e** Lysates of liver tissues were used for immunoblotting. Quantitative data represent mean ± standard error of the mean (SEM). *P < 0.05 and **P < 0.01

The global gene expression profile of patients with NASH from GSE24807 in the GEO database was analyzed to explore the molecular mechanism of NASH progression. The results showed a significant change in the expressions of 3678 genes compared with normal controls^22^. As expected, a number of well-known NASH-related genes, including *CXC motif chemokine ligand 10 (CXCL10)*, *SH3 domain–binding protein 5 (SH3BP5)*, *cytochrome P450 2R1 (CYP2R1)*, *fatty acid-binding protein 1 (FABP1)* and *transforming growth factor beta* 1 (*TGFB1*), were detected (Fig. 1a)^13,23–26^. Among the changed genes, the expression of a poorly defined gene, *ETS-1*, was significantly upregulated. It has been shown that the expression of Ets-1 was closely associated with TGF-β1 in the mouse epithelial cell^27^. Importantly, the database showed a consistent positive correlation between the expression levels of *TGFB1* and *ETS-1* in the liver tissues of patients with NASH (Fig. 1b).

Thus, C57/B6J mice were placed on an MCD diet to further investigate the association between Ets-1 and NASH. A histopathological examination of liver tissues was performed to demonstrate that the MCD diet induced key aspects of NASH pathology in mice (Supplementary Fig. S1A and S1B). Then, it is found that the expression of TGF-β1 was higher in the liver tissues of MCD diet-fed mice (Fig. 1c). In agreement, the expression of Ets-1 in the liver tissues of mice fed an MCD diet increased significantly, compared with that of mice fed a chow diet (Fig. 1d, e). These results raised the possibility that the upregulation of Ets-1 was linked to the progression of NASH.

### The expression of Ets-1 was upregulated by TGF-β1-Smad2/3

**Fig. 2:**
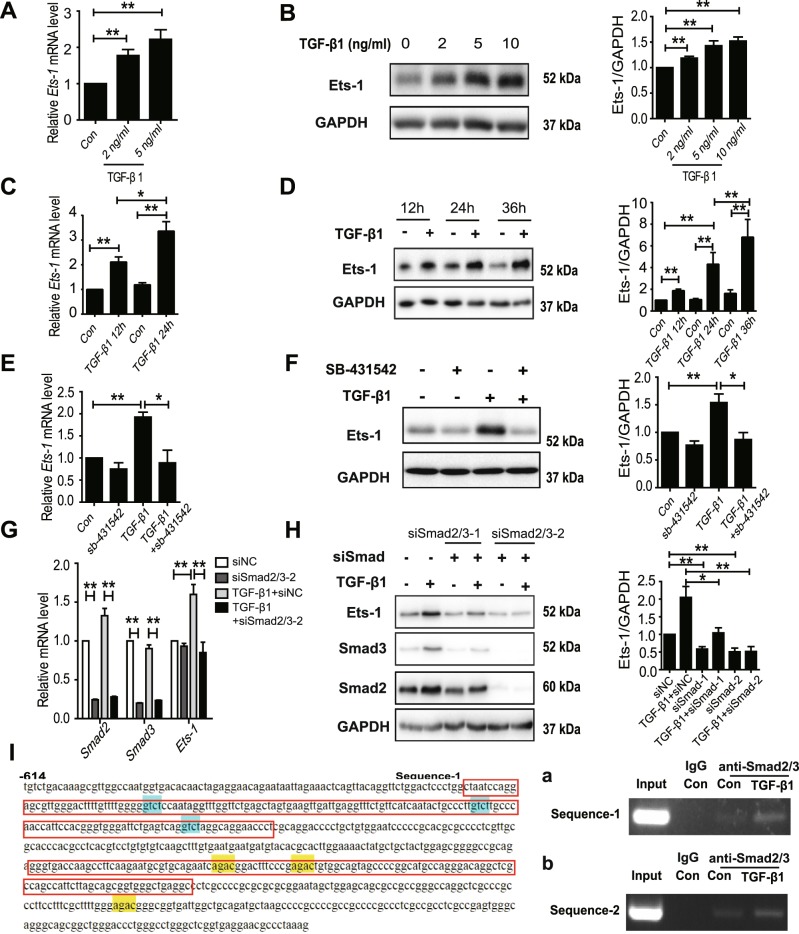
The expression of Ets-1 was directly regulated by TGF-β1-Smad2/3. **a**–**d** Primary hepatocytes from WT mice were (**a**, **b**) treated with different concentrations of TGF-β1 for 24 h or (**c**, **d**) harvested at different time points under the treatment of 10 ng/mL TGF-β1. **a**, **c** The relative mRNA level of *Ets-1* was measured using qPCR. **b**, **d** Immunoblots of Ets-1 are shown. **e**, **f** WT primary hepatocytes were pretreated with SB-431542 (10 μM) for 12 h. Then, 10 ng/mL TGF-β1 was added to the cells for 24 h. **e** The relative expression of *Ets-1* was measured using qPCR. **f** Immunoblots of Ets-1. **g**, **h** siRNAs of the negative control (siNC) and Smad2/3 (siSmad2/3-1 and siSmad2/3-2) were added to primary hepatocytes for 36 h, before incubation with 10 ng/mL TGF-β1 for 12 h (**g**) or 24 h (**h**). **g** The expression of *Smad2*, *Smad3*, and *Ets-1* were examined. **h** Immunoblotting for Smad3, Smad2, and Ets-1 was performed. **i** The promotor of *Ets-1* between –614 bp and –3 bp is shown. The locations of Sequence-1 and Sequence-2 are marked by a red box. The sequences of (C/AGAC) were Smad2/3-specific DNA-binding elements. **a**, **b** The ChIP assay of *Ets-1* promoter used primary hepatocytes treated with TGF-β1 (10 ng/mL) for 6 h. An anti-Smad2/3 polyclonal antibody was used for precipitation. PCR analysis of the input and immunoprecipitation with IgG and an anti-Smad2/3 antibody were performed. Quantitative data are expressed as mean ± SEM (at least three independent experiments). *P < 0.05 and **P < 0.01

Primary hepatocytes were treated with TGF-β1 to determine the relationship between TGF-β1 and Ets-1 in the liver, revealing that Ets-1 was dose-dependently upregulated by TGF-β1 (Fig. 2a, b). In addition, the expression of Ets-1 increased with the extension of TGF-β1 treatment (Fig. 2c, d). SB-431542, the selective inhibitor of ALK5 (TGF-β1 type I receptor), notably reversed the TGF-β1-induced elevation of Ets-1 in primary hepatocytes (Fig. 2e, f). It is shown that the complex formed by Smad proteins is an important conductor in TGF-β1 signaling. The TGF-β1-induced upregulation of Ets-1 was completely abolished by the interference of Smad2/3 and Smad4 (Fig. 2g, h, and Supplementary Fig. S2A).

A chromatin immunoprecipitation assay (ChIP) was conducted to explore whether the expression of Ets-1 was directly regulated by TGF-β1 signaling. The specific (C/AGAC) DNA-binding elements of Smad2/3 in the promoter of *Ets-1* between 0 and –2000 bp were screened. Two such element sequences were detected, and both were between –614 and –3 bp within the *Ets-1* promoter. Further results indicated that the binding of Smad2/3 to the 2 aforementioned regions was significantly enhanced in hepatocytes through TGF-β1 treatment (Fig. 2i). Moreover, a luciferase assay was conducted, leading to the discovery that the mutation of Smad2/3 binding sites impairs the luciferase activity of the *Ets-1* promoter region (Supplementary Fig. S2B). Together, these data showed that the elevation of the expression of Ets-1 in NASH model mice was directly regulated by TGF-β1-Smad2/3 in primary hepatocytes.

### Ets-1 inhibited the ubiquitination and proteasomal degradation of p-Smad3

**Fig. 3:**
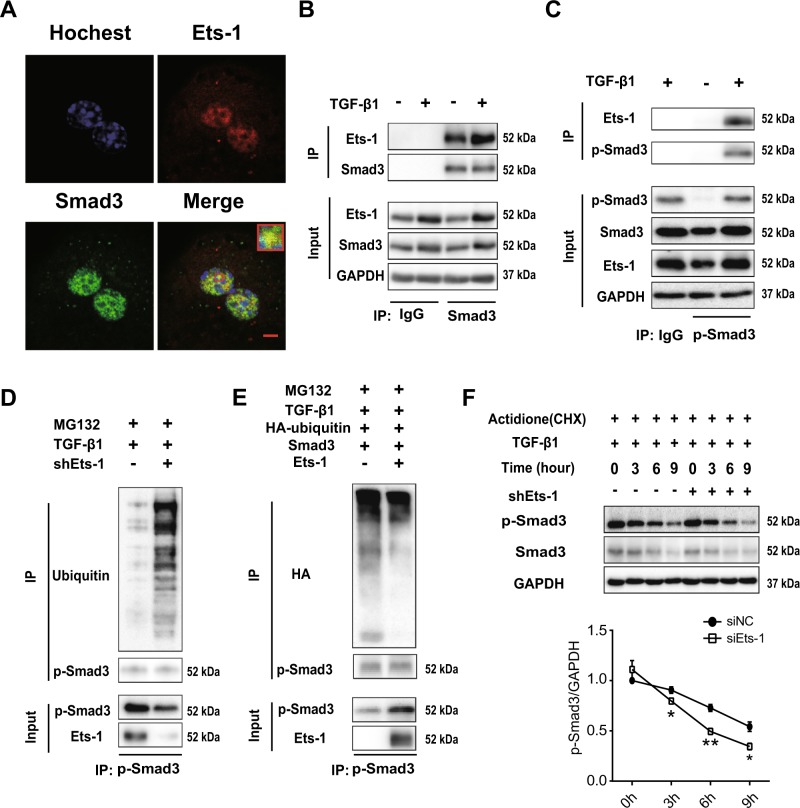
Ets-1 diminished the ubiquitination and proteasomal degradation of p-Smad3. **a** Primary hepatocytes were stained with Hoechst in blue, anti-Ets-1 antibody in red and anti-Smad3 antibody in green, followed by assessment using confocal microscopy. Scale bar: 5 μm. **b**, **c** Hepatocytes were treated with TGF-β1 (10 ng/mL) for 6 h. **b** Cell lysates were immunoprecipitated with an anti-Smad3 antibody, followed by immunoblotting with an anti-Smad3 or anti-Ets-1 antibody. **c** Total lysates of cells were subjected to co-IP analysis with an anti-phosphorylated Smad3 (p-Smad3) antibody and immunoblot of Ets-1 or p-Smad3. **d**, **e** Hepatocytes were transfected with shEts-1 adenovirus (**d**) or co-transfected with HA-ubiquitin, Smad3 and Ets-1 plasmids (**e**) for 24 h and then treated with TGF-β1 for another 2 h. Finally, MG132 (25 μM) was added for 4 h. The lysates were used for immunoprecipitation with p-Smad3, and immunoblot was examined using p-Smad3, anti-ubiquitin (**d**) or anti-HA (**e**) antibody. **f** Hepatocytes were infected with shNC and the shEts-1 adenovirus for 24 h and treated with TGF-β1 (10 ng/mL) for 2 h. Cycloheximide (CHX, 50 ng/mL) was added, and the cells were harvested for 3, 6 and 9 h. Immunoblotting for p-Smad3 and Smad3 were performed. Quantitative data represent mean ± SEM. *P < 0.05 and **P < 0.01

Ets-1 was found to bind to Smad3 in HeLa cells^28^. In primary hepatocytes, immunofluorescence experiments were performed to show a co-localization of Ets-1 and Smad3 in the nucleus (Fig. 3a). Then, a co-immunoprecipitation (co-IP) experiment was performed to detect an endogenous interaction between Ets-1 and Smad3. It is important to note that the interaction was strengthened in response to TGF-β1 treatment (Fig. 3b). Thus, further co-IP experiments were conducted, showing that Ets-1 binds to p-Smad3 in hepatocytes under TGF-β1 treatment (Fig. 3c). However, p-Smad2 did not bind to Ets-1 in hepatocytes (Supplementary Fig. S3A). It is shown that the protein stability of Smad3 can be regulated by directly binding with another transcriptional factor^29^. Ets-1 was not found to affect the ubiquitination of Smad3 in an inactive state (Supplementary Fig. S3B and S3C), however, the ubiquitination of p-Smad3, the active Smad3, was inhibited by Ets-1 in hepatocytes (Fig. 3d, e). Likewise, the half-life of p-Smad3 decreased significantly in Ets-1-deficient hepatocytes with TGF-β1 treatment (Supplementary Fig. S3D and Fig. 3).

The expressions of *ring-box* 1 (*Rbx1*)*, cullin 1* (*Cul1*) and *beta-transducin repeat containing protein* (*Btrc*) were examined to exclude the involvement of Ets-1 at the transcriptional level. Rbx1, Cul1, and Btrc are important components of ROC1-SCF (BTRC), which is the specific E3 ligase for Smad3. The mRNA levels of *Axin1* and *praja ring finger ubiquitin ligase 1* (*Pja1*), which encode the proteins specifically affecting the stability of Smad3^30,31^, were also detected. However, the expressions of the aforementioned genes showed no significant difference between negative control short hairpin RNA (shNC) and shEts-1 hepatocytes. Moreover, no change in the expressions of these genes was observed when hepatocytes were incubated with TGF-β1 (Supplementary Fig. S3E). In conclusion, this study showed that Ets-1 enhanced the stability of p-Smad3 in hepatocytes under TGF-β1 treatment.

### Ets-1 enhanced the activity of TGF-β1/Smad3 signaling

**Fig. 4:**
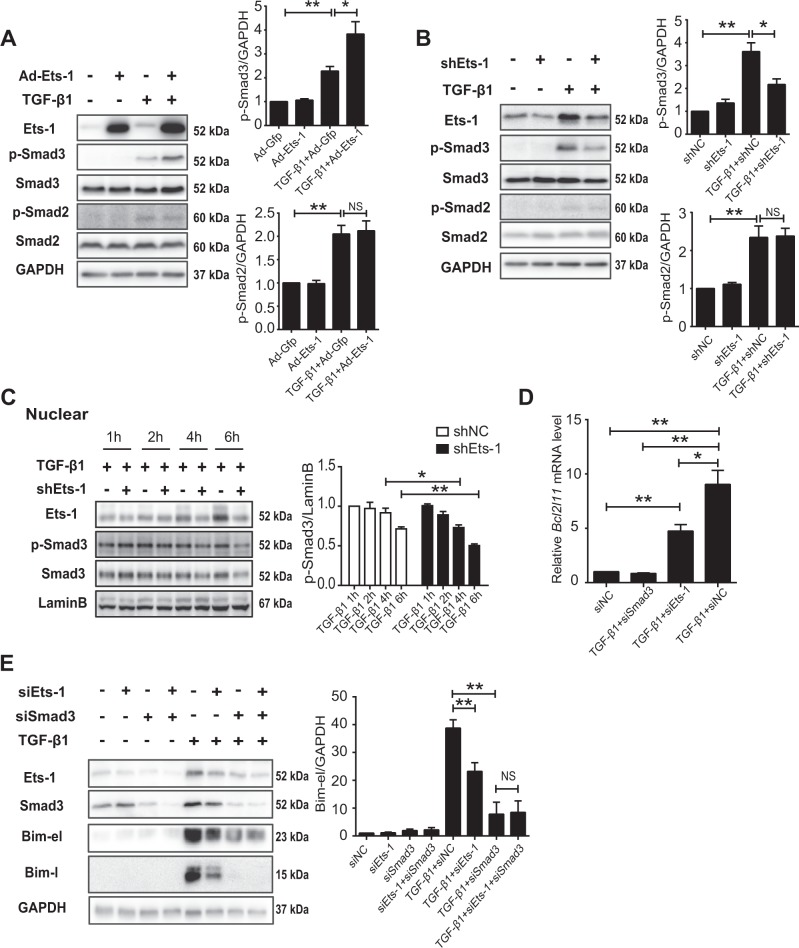
Ets-1 enhanced the activity of TGF-β1/Smad3 signaling. **a**, **b** Primary hepatocytes treated with Gfp and Ets-1 (**a**) or shNC and shEts-1 (**b**) recombinant adenovirus for 36 h. The cells were incubated with TGF-β1 (10 ng/mL) for 6 h, and the lysates were used for immunoblot analysis. (**c**) TGF-β1 (10 ng/mL) was added to hepatocytes for 1, 2, 4 and 6 h. The nuclear fraction was subjected to immunoblotting. **d**, **e** Primary hepatocytes were treated with siNC, siSmad3 or siEts-1 for 36 h and incubated with TGF-β1 (10 ng/mL) for 24 h. **d** mRNA level of *Bcl2l11* is shown. **e** Total lysates were used for immunoblotting of Ets-1, Smad3 and Bim. Quantitative data are presented as mean ± SEM (at least three independent experiments). NS (negative significance); *P < 0.05 and **P < 0.01

Given that the ubiquitination of p-Smad3 is important for modulating the activity of Smad3^32^, it was necessary to investigate whether Ets-1 participated in the transduction of TGF-β1 signaling. The overexpression of Ets-1 increased the TGF-β1-induced level of p-Smad3, but not of p-Smad2, in primary hepatocytes (Fig. 4a). Concordantly, Ets-1 deficiency impaired the level of p-Smad3. However, p-Smad2 remained unchanged under TGF-β1 treatment (Fig. 4b). Although Ets-1 is a transcriptional factor, the mRNA level of Smad3 was not altered by the downregulation or upregulation of Ets-1, even in the presence of TGF-β1 (Supplementary Fig. S4A and S4B).

As a transcriptional factor, Smad3 functions in the nucleus. This study detected that the decrease in nuclear p-Smad3 became more significant in Ets-1-deficient hepatocytes after 4 h of TGF-β1 treatment (Fig. 4c). Similarly, cytoplasmic p-Smad3 had a lower protein level in hepatocytes with Ets-1 knockdown in the presence of TGF-β1 (Supplementary Fig. S4C). Therefore, Ets-1 is beneficial in maintaining the protein level of p-Smad3 in the nucleus. The expression of *Bcl-2-like 11* (*Bcl2l11*), which was regulated by Smad3, was examined to further demonstrate that Ets-1 was critical in the regulation of Smad3 activity. The TGF-β1-induced upregulation of *Bcl2l11* was completely inhibited by the interference of Smad3. Moreover, Ets-1 deficiency significantly reversed the elevation of *Bcl2l11* (Fig. 4d), which was similar to another Smad3 downstream gene, *snail family zinc finger 1* (*Snai1*) (Supplementary Fig. S4D). Bim, encoded by *Bcl2l11*, has 3 isoforms: Bim-el, Bim-l and Bim-s. Consistently, the TGF-β1-induced elevation of Bim isoforms was abolished when Smad3 was knocked down, and it was effectively reversed by Ets-1 deficiency (Fig. 4e). Taken together, Ets-1 amplified the transcriptional activity of Smad3 in hepatocytes.

### Ets-1 accelerated hepatocyte apoptosis induced by TGF-β1/Smad3 signaling

**Fig. 5:**
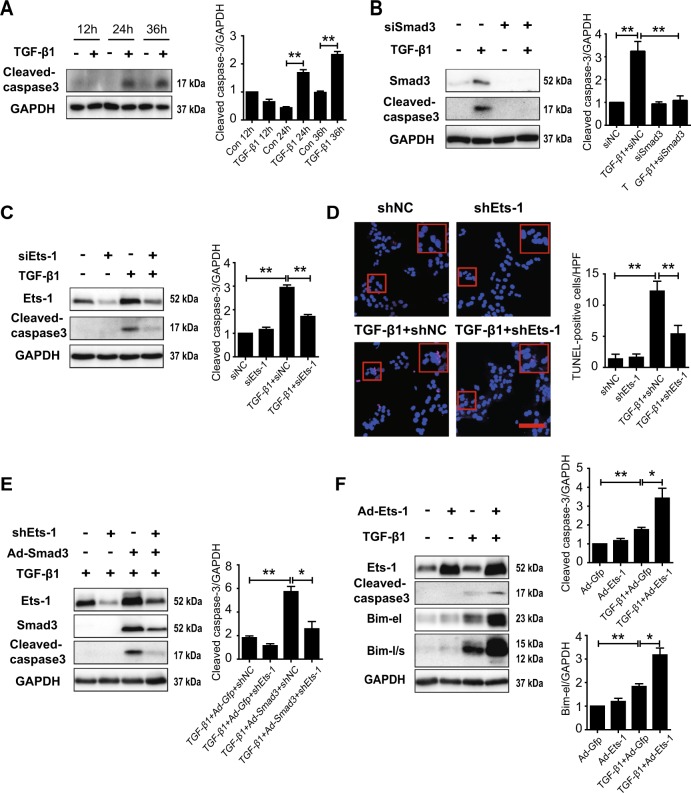
Ets-1 accelerated hepatocyte apoptosis induced by TGF-β1/Smad3 signaling. **a** Hepatocytes were treated with TGF-β1 (10 ng/mL) for 12, 24 and 36 h and then used for immunoblot analysis. **b**, **c** siRNAs of NC, Smad3 (**b**) or Ets-1 (**c**) were added to hepatocytes for 36 h and stimulated with TGF-β1 (10 ng/mL) for 24 h. Total lysates were subjected to immunoblotting of cleaved-caspase3, Smad3 (**b**) or Ets-1 (**c**). **d** Primary hepatocytes were treated with shNC and shEts-1 recombinant adenovirus for 36 h. Then, the cells were incubated with TGF-β1 (10 ng/mL) for 24 h. The cells were stained with TUNEL. Scale bar: 100 μm. Representative images are on the left, and quantitative analysis is on the right. **e** Hepatocytes were transfected with shNC or shEts-1 and Gfp or Smad3 recombinant adenovirus for 36 h. Then, the cells were stimulated with TGF-β1 (2 ng/mL) for 24 h. Lysates were used for immunoblotting. **f** Hepatocytes were transfected with Gfp and Ets-1 recombinant adenovirus for 24 h, and then TGF-β1 (2 ng/mL) was added for 36 h. Total lysates of cells were used for immunoblotting. Data are presented as mean ± SEM (at least three independent experiments). *P < 0.05 and **P < 0.01

Elevated Bim has been shown to facilitate apoptosis in hepatic cell lines^33^. Consequently, this study found that the protein level of cleaved caspase-3, a biomarker for apoptosis, increased when primary hepatocytes were treated with TGF-β1 (Fig. 5a). The knockdown of Smad3 was able to abolish a TGF-β1-induced increase in cleaved caspase-3 in primary hepatocytes (Fig. 5b). However, Ets-1 deficiency also inhibited an increase in cleaved caspase-3 (Fig. 5c). In support of this, the results of the terminal deoxynucleotidyl transferase-mediated dUTP nick end labeling (TUNEL) and Annexin V staining assays further demonstrated that the lack of Ets-1 decreased TGF-β1-induced hepatocyte apoptosis (Fig. 5d and Supplementary Fig. S5A).

Although overexpressed Smad3 alone could not induce hepatocyte apoptosis (Supplementary Fig. S5B), a Smad3 abundance could enhance hepatocyte apoptosis when a low dose of TGF-β1 was used. Furthermore, the exacerbation of hepatocyte apoptosis was also weakened by Ets-1 knockdown (Fig. 5e). Concordantly, the forced expression of Ets-1 reinforced the effect of TGF-β1, and it increased the expressions of Bim and cleaved caspase-3 in hepatocytes (Fig. 5f). These results demonstrated that Ets-1 participated in apoptosis, mediated by TGF-β1/Smad3 signaling, which promoted the progression of NASH.

### Ets-1 knockdown alleviated MCD diet-induced NASH in mice

**Fig. 6:**
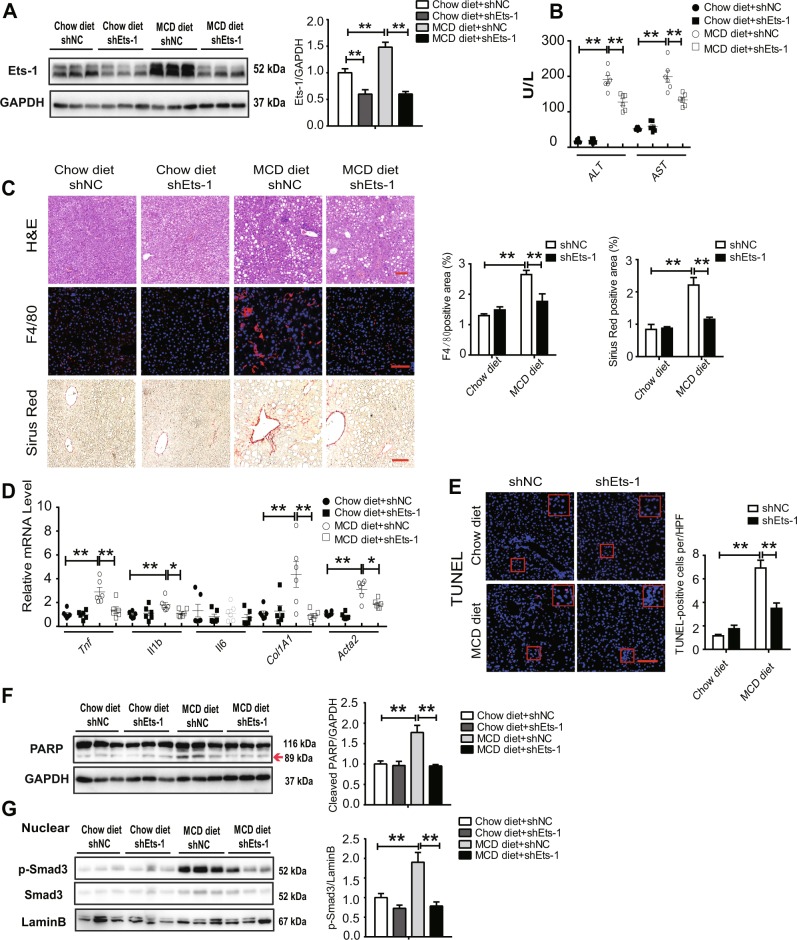
Ets-1 was elevated in the liver tissues of NASH mice. WT mice were injected with AAV8-shNC virus and AAV8-shEts-1 virus through the tail vein for 2 weeks and divided into four groups: chow diet+shNC (*n* = 6), chow diet+shEts-1 (*n* = 6), MCD diet+shNC (*n* = 6), and MCD diet+shEts-1 (*n* = 6). The mice were fed with a chow diet or an MCD diet for 8 weeks and sacrificed. **a** The lysates of liver tissues were used for immunoblotting of Ets-1. **b** ALT and AST levels in serum, U/L: enzyme activity per liter of liquid. **c** Liver sections were stained with H&E, Sirius Red, and F4/80. Scale bar: 100 μm. The left panel is a representative image, and the right panel is the quantification of Sirius Red–positive and F4/80-positive areas. The results were quantified using the Image J software. **d** Hepatic mRNA levels of inflammation-related proteins (*Tnf*, *Il1b* and *Il6*) and fibrosis-related proteins (*Col1A1* and *Acta2*) were quantified using qRT-PCR. **e** Liver specimens were stained with TUNEL. A representative image is shown on the left, with the corresponding statistical result on the right. **f**, **g** Total lysates (**f**) or nuclear lysates (**g**) of liver tissues were used for immunoblotting. The left panels are representative images, and the right panels are corresponding statistical results. The red arrow is referring to cleaved PARP. Quantitative data represent mean ± SEM. *P < 0.05; **P < 0.01

Adeno-associated virus type 8 (AAV8)-containing shNC or shEts-1 were injected into the tail veins of mice to determine whether Ets-1 contributed to NASH progression in mice fed an MCD diet. Following 8 weeks of the MCD diet, the study showed that the upregulation of the expression of Ets-1 was reversed by shRNA in liver tissues (Fig. 6a). Despite no significant change in hepatic triacylglycerol (TAG), serum non-esterified fatty acid (NEFA), or serum cholesterol between shNC and shEts-1 mice (Supplementary Fig. S6A, S6B and S6C), the levels of serum alanine aminotransferase (ALT) and aspartate aminotransferase (AST), markers of liver injury, decreased notably when Ets-1 was knocked down (Fig. 6b). Moreover, hematoxylin and eosin (H&E) staining, F4/80 immunofluorescence staining, and Sirius Red staining of liver sections showed that the downregulation of Ets-1 significantly ameliorated liver fibrosis and inflammation induced by the MCD diet (Fig. 6c). Consistently, the expressions of genes associated with inflammation (*Tnf*, *Il1b*, and *Ccl2*) and fibrosis (*Col1A1*, *Acta2*, and *Timp1*) decreased significantly (Fig. 6d and S6D). The number of MCD diet-induced TUNEL-positive hepatocytes reduced markedly (Fig. 6e). In addition, the protein level of cleaved poly ADP ribose polymerase (PARP) decreased significantly in liver tissues of Ets-1-downregulated mice (Fig. 6f). In agreement, the knockdown of Ets-1 led to a decrease in p-Smad3 in the nuclei of cells from the liver tissues of mice fed an MCD diet (Fig. 6g). Collectively, these data indicated that the knockdown of Ets-1 significantly alleviated hepatocyte apoptosis induced by the MCD diet and improved NASH in mice.

## Discussion

**Fig. 7:**
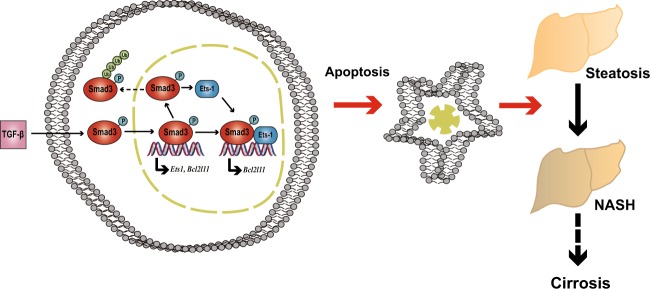
Working model: Ets-1 maintained nuclear p-Smad3 and enhanced hepatocyte apoptosis induced by TGF-β1, thereby promoting NASH progression

Previous reports have shown that hepatocyte apoptosis is critical to the progression of NASH^3,8^. However, the mechanisms resulting in persistently increased apoptosis in NASH remain poorly understood. This study demonstrated that the expression of Ets-1 was upregulated by TGF-β1 signaling, and it was required for maintaining p-Smad3 in the nucleus. Continuously high levels of nuclear p-Smad3 induced hepatocyte apoptosis and eventually accelerated the progression of NASH (Fig. 7).

Ets-1, generally viewed as a transcription factor, is widely expressed in multiple cells and tissues^34^. Although Ets-1 participates in apolipoprotein F transcription, the exact function of Ets-1 in hepatocytes remains unknown^35^. This study investigated whether Ets-1 was directly regulated by Smad2/3 in primary hepatocytes. It showed a Smad2/3 binding site within the *ETS-1* promoter region in the HaCaT cell line^36^. The bioinformatics analysis revealed that the sequences of the *Ets-1* promoter were conserved across different species, and one of the detected sequences was similar to the identified binding site in the HaCaT cell line. Importantly, the study confirmed that the sites were crucial for the upregulation of Ets-1 by TGF-β1 signaling in hepatocytes.

Smad3 is downstream of TβRI and mediates the transduction of TGF-β1 signaling by stimulating the expression of genes in the nucleus. However, the affinity between Smad3 and DNA is weak, hence, other interacting proteins are needed^37,38^. Among the partners, Ets-1 is generally viewed as a transcriptional activator that recruits Smad3 to specific sites. For example, Ets-1 cooperates with Smad3 to stimulate the expression of parathyroid hormone-related protein (PTHrP) in breast cancer cells^39^. However, this study showed that Ets-1 directly bound to p-Smad3 and maintained the activation of Smad3 by diminishing ubiquitination and proteasomal degradation. Coincidentally, it was reported that another transcription factor, B-cell lymphoma 3 (Bcl-3), also inhibited the degradation of Smad3 through direct binding, but not through transcriptional activity^29^. Despite this, further assays are needed to clarify whether Ets-1 stabilizes the DNA binding of p-Smad3 or simply functions as a coupling protein that protects p-Smad3 from degradation in hepatocytes.

Apart from the ubiquitination degradation of p-Smad3, the phosphorylation of Smad3 is also important for the activity of TGF-β1/Smad3 signaling^30^. This study found that the expression of nuclear p-Smad3 did not differ between control and Ets-1-deficient hepatocytes in the early stage of TGF-β1 treatment. Moreover, the transcriptional expression of *Ppp2ca*, which encodes protein phosphatase 2A (PP2A), which is the specific phosphatase for Smad3^32^, did not change when Ets-1 was knocked down. Thus, Smad3 phosphorylation might not be affected by Ets-1. In addition, this study primarily focused on the effect of Ets-1 on the degradation of p-Smad3. Ets-1 also bound to Smad3. Since exposure in TGF-β1 led to an upregulation of Smad3 in primary hepatocytes, we adjusted the amount of Smad3 to the same in the IP experiment and observed that the binding affinity of Ets-1 and Smad3 was enhanced by TGF-β1 treatment. However, the degradation of Smad3 was not obvious when Ets-1 was knocked down. Although further research is needed to determine the mechanisms involved, the effect of Ets-1 on p-Smad3 might contribute to the TGF-β1-induced upregulation of the total protein level of Smad3.

Smad2 and Smad3 share protein sequence similarities of 92%, however, both proteins have different functions^40^. A previous study reported that Smad3, not Smad2, was the key mediator of TGF-β1-induced apoptosis in Hep3B hepatoma cells^33^. Additionally, pan-caspase inhibitors were shown to effectively block hepatocyte apoptosis and attenuate liver injury, inflammation, and fibrosis^9,41^. Apparently, hepatocyte apoptosis, a well-recognized programmed cell death, is important for the progression of NASH^42,43^. This study confirmed that the forced expression of Smad3 could enhance hepatocyte apoptosis induced by TGF-β1. Moreover, a further increase in hepatocyte apoptosis was controlled when Ets-1 was knocked down. Our previous study showed that Ets-1 was critical in protecting β cells from hypoxia-induced apoptosis^19^. Meanwhile, Wang et al. showed that the silencing of Ets-1 was beneficial for cardiomyocyte survival in the face of glucotoxicity^21^. These conflicting results indicated that Ets-1 exerted different functions in response to apoptosis caused by different stimuli.

Besides decreased hepatocytes apoptosis, the MCD diet-induced activation of TGF-β1/Smad3 signaling diminished in the liver tissues of Ets-1 knocked down mice. It showed that the accumulation of liver fat, which was induced by a high-fat diet, reduced in the livers of Smad3-knockout mice. However, the insulin sensitivity of liver tissues did not increase. The decrease in fat in the liver was found to be caused by the suppressed differentiation of white adipose tissue^44,45^. These data showed that Smad3 might not be important for the metabolism of fat in the liver. Likewise, no obvious reduction in TAG was found in the liver tissues of mice with lower expressions of Ets-1. Altogether, the knockdown of Ets-1 significantly alleviated hepatocyte apoptosis, protecting the liver from injury, inflammation, and fibrosis in the NASH mouse model.

Similar to *Bcl2l11*, the expression of *Snai1*, which was also regulated by TGF-β1/Smad3 signaling at the transcriptional level^46^, could be inhibited by Ets-1 knockdown (Supplementary Fig. S4D). By suppressing the expression of E-cadherin, Snai1, encoded by *Snai1*, had an effect on the transdifferentiation of hepatocytes to mesenchymal cells in the process of epithelial-to-mesenchymal transition, thus aggravating liver fibrosis^47,48^. In addition, TGF-β1/Smad3 signaling participated in the activation of HSCs by promoting the expressions of type I and type III collagen^12^. In HSCs, Ets-1 was shown to modulate the mRNA expression of *connective tissue growth factor* (*Ctgf*), which encodes a pro-fibrotic factor. However, whether or not Ets-1 contributes to the activation of HSCs is unclear^49^. Accordingly, future studies on the role of Ets-1 in liver fibrosis may be of great significance.

To summarize, Ets-1 acts as a positive regulator of TGF-β1 signaling, which accelerates hepatocyte apoptosis and the progression of NASH. Although further studies are needed to elucidate the mechanism, the findings of this study indicated that Ets-1 might serve as a new target in the pathogenesis of NASH, thus providing a basis for the design of new strategies to cure this disease.

## Materials and Methods

### Animals

Male 6-week-old C57/B6J mice were purchased from Nanjing University, China. Then, 100 μL of 1 × 10^13^ virus granule (VG)/mL AAV8-shNC virus or AAV8-shEts-1 virus (Obio, Shanghai, China) was injected into the tail veins of mice for 2 weeks. Then, the mice were fed with chow diet and MCD diet (Research Diets, NJ, USA) for 8 weeks and sacrificed without fasting. After administering isoflurane anesthesia, blood was collected from the vena cava and the serum was isolated. The tissues from the left lateral lobes of the livers were fixed in 4% (wt/vol) paraformaldehyde for 2 h, transferred to 20% (wt/vol) sucrose, and embedded in paraffin the next day. Other parts of the tissues were snap-frozen in liquid nitrogen. The animal experiments were approved by the Institutional Animal Care and Use Committee of Nanjing Medical University.

### Measurement of serum chemistry

The activities of ALT and AST were examined using the Alanine/Aspartate Aminotransferase Reagent Kit from Shensuo UNF (Shanghai, China). The hepatic triglyceride, NEFA and cholesterol levels were analyzed as described in a previous study^50^.

### H&E, Sirius Red, F4/80, and TUNEL staining of mouse liver tissues

The liver tissues were cut into 5-μm sections for H&E, Sirius Red, F4/80 (Proteintech, IL, USA), and TUNEL staining (Vazyme, Jiangsu, China). The F4/80- and Sirius Red-positive areas were quantified using the ImageJ software. TUNEL-positive cells were counted on 10 high-power fields (HPF)/slide.

### NASH Clinical Research Network Histologic Scoring System

The liver sections of mice were assessed using the NAFLD activity score, which ranged from 0 to 8 and was a sum of three histologic scores, such as steatosis (0–3), lobular inflammation(0–3), and ballooning degeneration (0–2)^51^.

### Reagents and antibodies

SB-431542 was purchased from Selleckchem (TX, USA). Rabbit anti-Ets-1, the Smad2/3 Antibody Sampler Kit, rabbit anti-Bim, rabbit anti-PARP, rabbit anti-cleaved caspase-3 and horseradish peroxidase–conjugated anti-mouse secondary antibodies were purchased from Cell Signaling Technology (MA, USA). Goat polyclonal antibody Lamin B and mice monoclonal antibody HA-probe were purchased from Santa Cruz Biotechnology (CA, USA). The rabbit anti-GAPDH antibody was purchased from Bioworld Technology (MN, USA). IPKine™ HRP, Goat Anti-Rabbit IgG LCS was obtained from Abbkine (Wuhan, Hubei, China). Cycloheximide (CHX) was obtained from Sigma (MO, USA). The nuclear and cytoplasmic proteins of liver tissues and hepatocytes were extracted using a reagent kit from KeyGen Biotech (Nanjing, Jiangsu, China).

### Plasmid construction and luciferase assay

The mouse Smad3 expression plasmid pCMV5-Smad3 was constructed by inserting the full-length coding region of Smad3 (NM_016769.4) into the pCMV5 vector. The HA-ubiquitin plasmid was constructed by inserting a single ubiquitin coding sequence into pRKS-HA.

The wide-type luciferase reporter plasmid (WT) was constructed by inserting a 725 bp sequence from -721 to 3 bp on the *Ets-1* promoter region into the pGL3-Basic vector (Promega, USA), while the mutation luciferase reporter plasmid (MT) was produced by mutating ‘agac’ to ‘attc’ and ‘gtct’ to ‘gaat’ in the insertion sequence.

In the luciferase assay, primary hepatocytes were co-transfected with WT/pCMV5, WT/Smad3, MT/pCMV5, MT/Smad3 and Renilla plasmids for 48 h. Luciferase activity was determined by the Dual-Glo^®^ Luciferase Assay System (Promega, USA) on the TE-20/20 Luminometer (Turner BioSystems, USA).

### Primary hepatocyte isolation and treatment

The hepatocytes were isolated from male 8-week-old C57/B6J mice fasted for 6 h. The viability of freshly isolated cells was examined using trypan blue (Sigma, MO, USA) ensuring more than 90% viability. After the hepatocytes were attached to dishes in DMEM-low glucose (Sigma) plus 10% (vol/vol) FBS (Gibco, ON, USA) for 4 h, the medium was changed to DMEM-low glucose containing 0.1% bovine serum albumin (BSA) (wt/vol) (Sigma) for further studies on the same day. The hepatocytes were incubated with the control, overexpressed Smad3 (ViGene, Shandong, China), overexpressed Ets-1, shNC and shEts-1 adenovirus with 50 MOI or transduced with the negative control, Smad4 and Smad2/3-specific small interfering RNA (siRNA) (RiboBio, Guangdong, China) using Lipofectamine 2000 (Invitrogen, NY, USA). The siRNA sequences used for analysis are listed in Supplementary Table S1.

### Quantitative real-time polymerase chain reaction

Total RNA was extracted using TRIzol reagent (Invitrogen) following the manufacturer’s protocol. An equal amount of total RNA was reverse transcribed using ReverTra Ace-α-reagent (Toyobo, Osaka, Japan), and the cDNA was mixed with the SYBR Green Real-time Polymerase Chain Reaction (PCR) Master Mix (Toyobo) and used for qPCR analysis on the LighteCycler480 II Sequence Detection System (Roche, Basel, Switzerland). The expression of ribosomal protein 36B4 was used as an internal standard. The primer sequences used for analysis are listed in Supplementary Table S2.

### Immunoblotting

Primary hepatocytes and liver tissues were lysed using ice-cold lysis buffer containing 50 mM Tris-HCl (pH 7.4), 150 mM NaCl, 0.1% SDS, 0.02% sodium azide, 1% NP-40, 1% deoxycholic acid sodium salt and 1 ug/mL proteinase inhibitors (Roche, IN, USA). The gel was blotted to a polyvinylidene difluoride membrane (Merck, CA, USA). The proteins were examined using the specific antibodies described earlier, visualized by chemiluminescence, and recorded using the ChemiQ 4800mini (CLINX, Shanghai, China). The relative protein levels were quantified using the ImageJ software.

### Flow cytometry analysis of apoptosis

Primary hepatocytes were treated using 10 ng/mL TGF-β1 (PeproTech, NJ, USA) for 12 h and cells were stained with Annexin V (annexin V-FITC apoptosis detection kit I, BD Biosciences, USA) according to the manufactures’ protocols. In total, 1 × 10^5^ cells in each sample were measured by a FACSCalibur flow cytometer and the Cellquest Pro software (BD Biosciences, USA).

### Chromatin immunoprecipitation

Primary hepatocytes were incubated with 10 ng/mL TGF-β1 for 6 h and then cross-linked with 1% formaldehyde for 10 min at 37 °C. The SDS lysis buffer was added and the cells were sonicated to shear DNA to lengths between 200 and 1000 base pairs. The chromatin was incubated with antibodies against Smad2/3 and IgG at 4 °C overnight. ChIP-enriched DNA was used as the template for PCR and the products were run in an agarose gel to detect the enrichment of Smad2 and Smad3 on the promoter of Ets-1. The primers used for examination are listed in Supplementary Table S3.

### Immunoprecipitation and ubiquitination analysis

Primary hepatocytes were treated with TGF-β1 for 6 h and then lysed in a buffer, as described in a previous study^19^. For the ubiquitination assay, the hepatocytes were first transfected with shRNA adenovirus or plasmids using effectene transfection reagent (Qiagen, Germany) for 24 h, stimulated with TGF-β1 for 2 h, and finally treated with carbobenzoxy-Leu-Leu-leucinal (MG132, Sigma, USA) for 4 h. The lysates were incubated with antibodies against Smad3, p-Smad3 or p-Smad2 overnight, and then protein A/G beads (Roche, Switzerland) were added for 2 h. The precipitates were boiled for 5 min and were examined using immunoblot with mouse anti-Ets-1 (Santa Cruz, USA) or mouse anti-ubiquitin antibody (CST, MA, USA).

### Immunofluorescence

Primary hepatocytes were cross-linked with 4% paraformaldehyde at room temperature for 30 min and then incubated with 0.2% Triton X-100 for 5 min. TUNEL staining was performed using the BrightRed Labeling Mix (Vazyme, Jiangsu, China). For immunofluorescence, 5% BSA was used for blocking, and the cells were labeled with mouse anti-Ets-1 antibody and anti-Smad3 antibody overnight at 4 °C. The next day, the samples were incubated with fluorophore-conjugated secondary antibody (Invitrogen) for 1 h at room temperature and then viewed on an Olympus FV1200 Laser Scanning Microscope.

### Public data

The gene expression profiles of liver tissues from patients with NASH were based on the GSE24807 database, which was obtained from the National Center for Biotechnology Information GEO database.

### Statistical analysis

The data were presented as mean ± standard error of the mean (SEM) and analyzed using the SPSS software. The differences between two groups were analyzed using the Student *t* test. The one-way analysis of variance (ANOVA) with post-hoc Tukey’s test was used to evaluate the differences among groups when three or more groups were analyzed. A *P* value less than 0.05 was considered statistically significant.

## Supplementary information

Supplementary Information
