# Supplementary material for: Ets-1 deficiency alleviates nonalcoholic steatohepatitis via weakening TGF-β1 signaling-mediated hepatocyte apoptosis
Source: Cell Death Dis. 2019 Jun 12;10(6):458. doi: 10.1038/s41419-019-1672-4 (PMC6561928; doi:10.1038/s41419-019-1672-4)
Supplement: Supplementary file 1 — Supplementary Information [file 41419_2019_1672_MOESM1_ESM.docx]

**Supplementary Information**


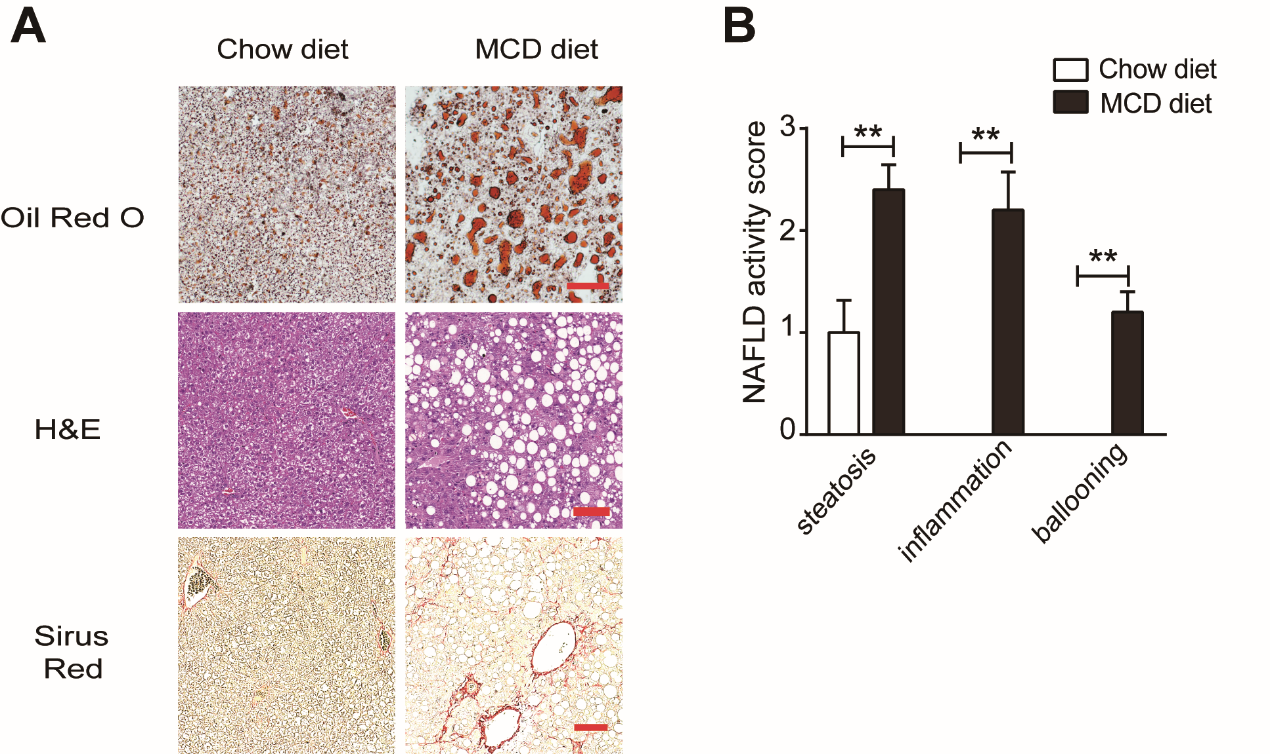


**Supplemental Figure S1.** WT mice were fed on a Chow diet or an MCD diet for 8 weeks and sacrificed. (A) Liver sections were stained with Oil Red O, H&E, Sirius Red. (B) Liver specimens were assessed by NAFLD activity score. Quantitative data represent mean ± standard error of the mean (SEM). **P* ＜ 0.05 and ***P* ＜ 0.01.


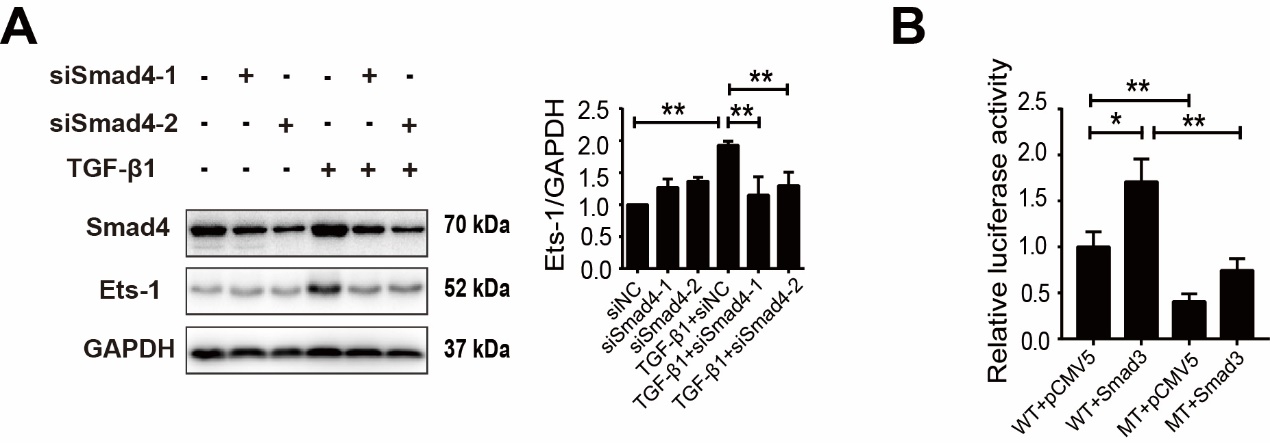


**Supplemental Figure S2.** (A) Hepatocytes were transfected with siRNA of Smad4 (siSmad4-1 and siSmad4-2) for 36h and then incubated with TGF-β1 (10 ng/ml) for 24h. Total lysates were used for immunoblot analysis. (B) The luciferase reporter assay was carried out in hepatocytes co-transfected with wide-type (WT) reporter plasmids, mutation (MT) reporter plasmids, control plasmids (pCMV5) and Smad3 plasmids. Quantitative data represent mean ± standard error of the mean (SEM). **P* ＜ 0.05 and ***P* ＜ 0.01.


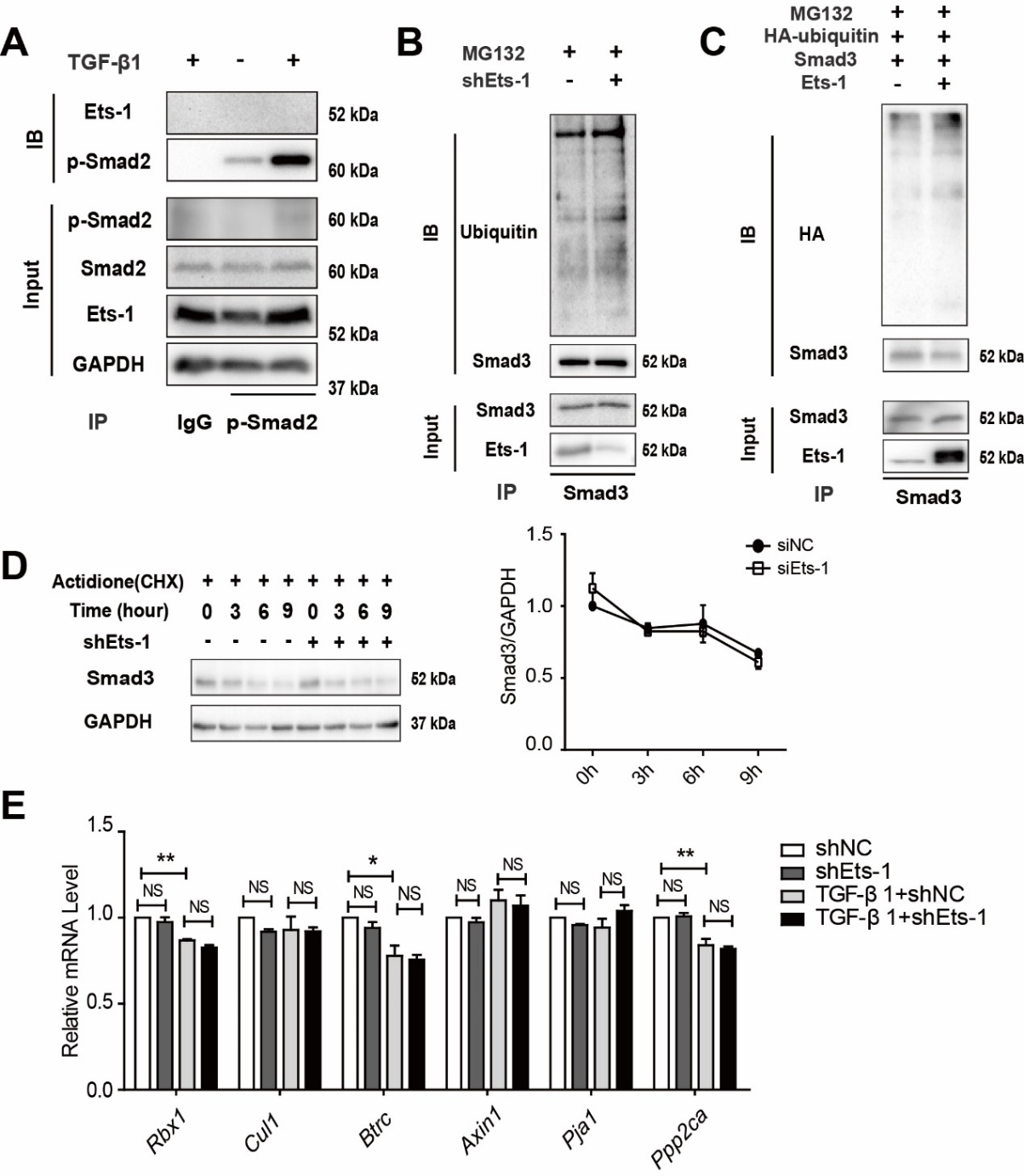


**Supplement****al Figure S3.** (A) Hepatocytes were treated with TGF-β1 to examine the interaction between Ets-1 and p-Smad2. (B and C) Hepatocytes were transfected with shEts-1 adenovirus (B) or co-transfection with HA-ubiquitin, Smad3 and Ets-1 plasmids (C) for 24h and an anti-Smad3 antibody was subjected to co-IP analysis. The lysates were immunoblotted with anti-ubiquitination (B) or anti-HA (C) antibodies. (D) Hepatocytes were transfected with shNC or shEts-1 adenovirus to detect the degradation of Smad3. (E) The mRNA levels of *Rbx1*, *Cul1*, *Btrc*, *Axin1*, *Pja1* and *Ppp2ca* were detected. Quantitative data are presented as mean ± SEM. NS (negative significance); **P* ＜ 0.05 and ***P* ＜ 0.01.


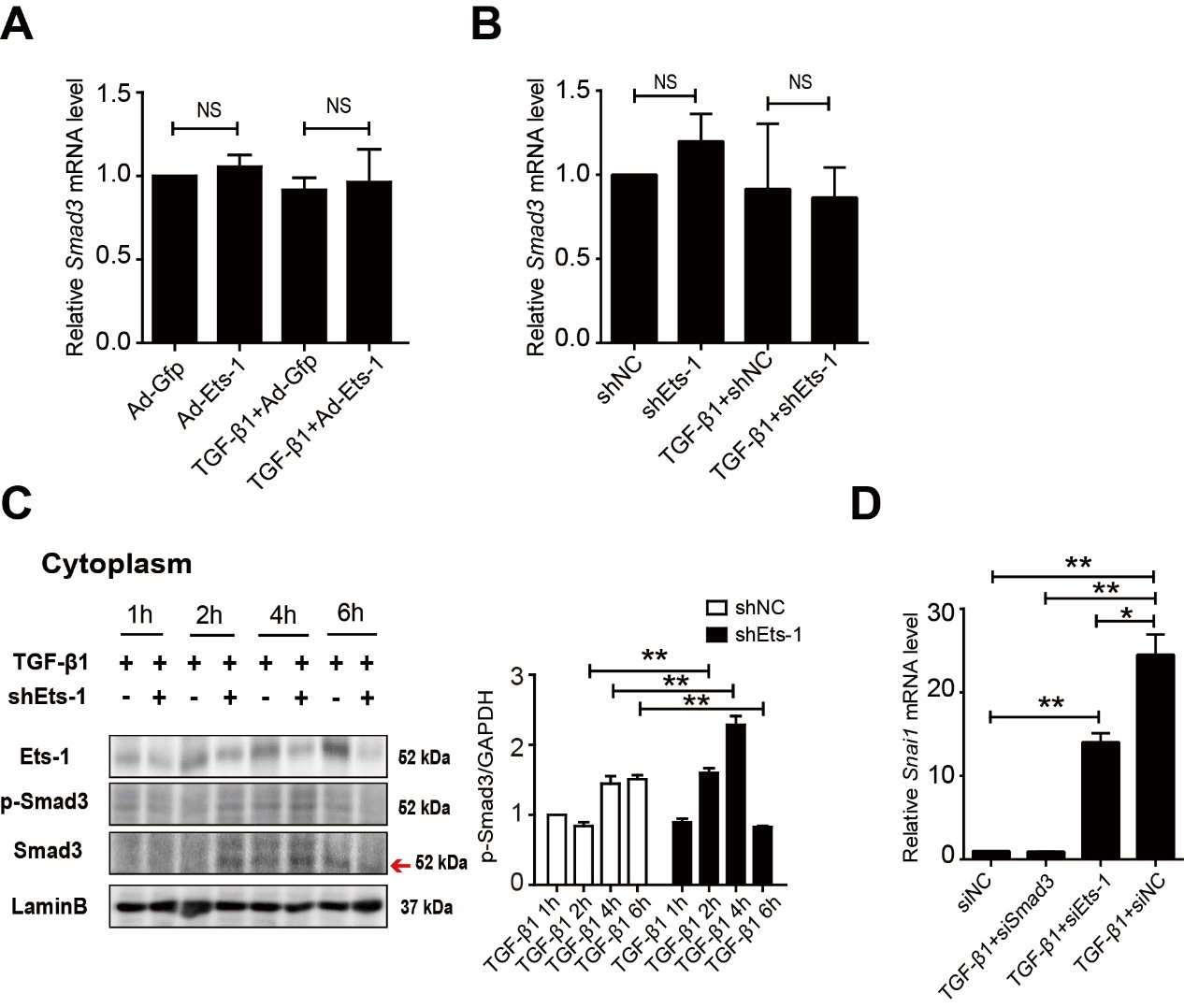


**Supplemental Figure S4.** (A and B) Upregulation or downregulation of Ets-1 were subjected to examine the expression of *Smad3*. (C) Cytoplasmic Ets-1 and p-Smad3 were detected in Ets-1-deficient hepatocytes. The red arrow is referring to Smad3. (D) The expression of *Snail1* was examined in hepatocytes with interference of Smad3 or Ets-1 under the treatment of TGF-β1. Quantitative data are presented as mean ± SEM. NS (negative significance); **P* ＜ 0.05 and ***P* ＜ 0.01.


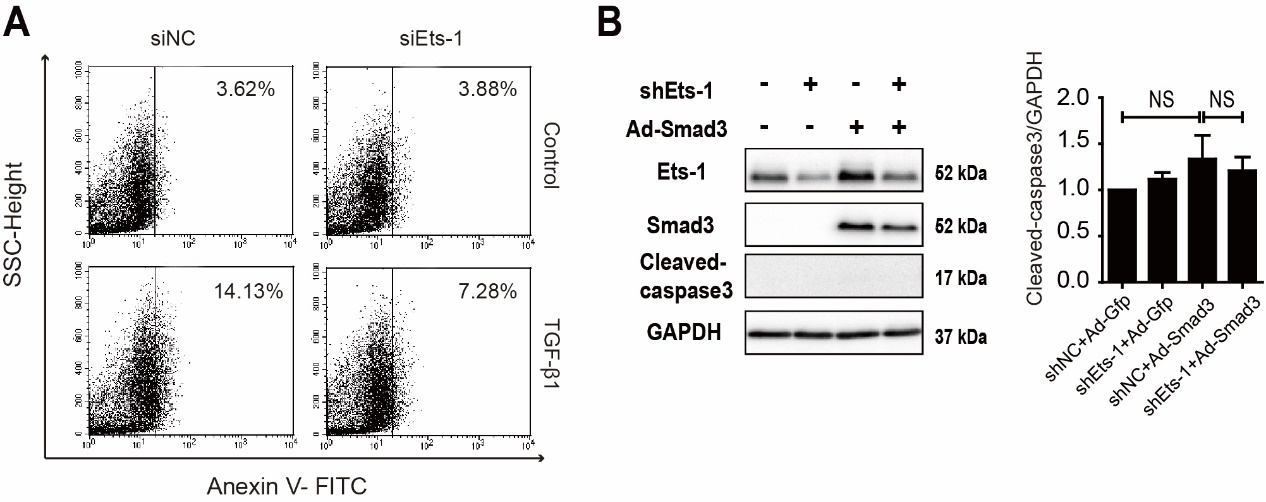


**Supplemental Figure S5.** (A) The Annexin V staining assays were used to examine the apoptosis induced by TGF-β1 for 12 h following the expression of Ets-1 was downregulated by siRNA in hepatocytes. (B) Hepatocytes were treated with Smad3 or shEts-1 adenovirus to examine the hepatocyte apoptosis**.** Quantitative data are presented as mean ± SEM. NS (negative significance); **P* ＜ 0.05 and ***P* ＜ 0.01.


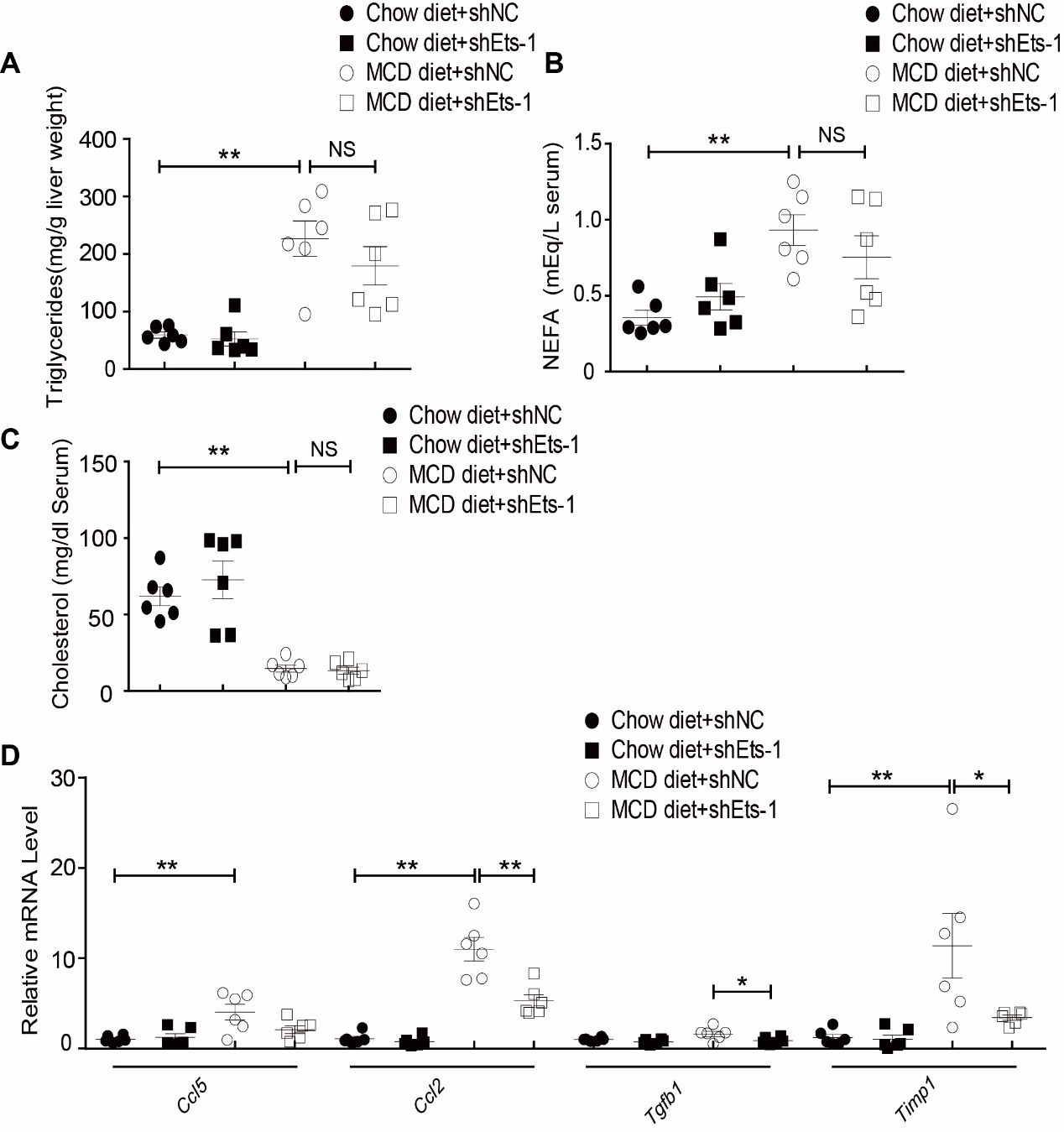


**Supplemental Figure S6.** WT mice were injected with AAV8-shNC virus or AAV8-shEts-1 virus for 2 weeks and then fed with an MCD diet for 8 weeks. (A) Hepatic triacylglycerol was detected. (B-C) The non-esterified fatty acid (NEFA) (B) and Cholesterol (C) in serum were detected. (D) Hepatic mRNA levels of inflammation related proteins (*Ccl5* and *Ccl2*) and pro-fibrotic proteins (*Tgfb1* and *Timp1*) were quantified using qRT-PCR. Quantitative data are presented as mean ± SEM. NS (negative significance); **P* ＜ 0.05 and ***P* ＜ 0.01.

**Table S1.** The sequences of siRNA used in experiments.

| NAME | SEQUENCES(5'-3') |
| --- | --- |
| siSmad2/3-1 | TGAAGATCTTCAACAACCA |
| siSmad2/3-2 | GGATGAAGTGTGTGTAAAT |
| siSmad4-1 | GATGAATTGGATTCTTTAA |
| siSmad4-2 | GGATGAGTACGTTCACGAC |

**Table S2.** The primer sequences used in qRT-PCR studies.

| NAME | SEQUENCES(5'-3') | |
| --- | --- | --- |
|  | Forward | Reverse |
| Ets-1 | GCTCAGTGTGTTCCTCCCTC | GCTGATAGGATGCAGCGTCT |
| Tgfb1 | GACCCTGCCCCTATATTTGGA | CCGGGTTGTGTTGGTTGTAGA |
| Col1A1 | CGTCTGGTTTGGAGAGAGCAT | GGTCAGCTGGATAGCGACATC |
| Acta2 | CTGACAGAGGCACCACTGAA | CATCTCCAGAGTCCAGCACA |
| IL-6 | AGTTGCCTTCTTGGGACTGA | TCCACGATTTCCCAGAGAAC |
| TNF-α | CTGAGGTCAATCTGCCCAAGTAC | CTTCACAGAGCAATGACTCCAAAG |
| IL-1β | CAACCAACAAGTGATATTCTCCATG | GATCCACACTCTCCAGCTGCA |
| Smad2 | ATGTCGTCCATCTTGCCATTC | AACCGTCCTGTTTTCTTTAGCTT |
| Smad3 | CACGCAGAACGTGAACACC | GGCAGTAGATAACGTGAGGGA |
| Bim | CCCGGAGATACGGATTGCAC | GCCTCGCGGTAATCATTTGC |
| Ccl2 | AGGTCCCTGTCATGCTTCTG | TCTGGACCCATTCCTTCTTG |
| Ccl5 | TGCCCACGTCAAGGAGTATTT | TTCTCTGGGTTGGCACACACT |
| Timp-1 | CGAGACCACCTTATACCAGCG | ATGACTGGGGTGTAGGCGTA |
| 36B4 | CACTGGTCTAGGACCCGAGAAG | GGTGCCTCTGGAGATTTTCG |

**Table S3.** The primer sequences used in ChIP studies.

| NAME | SEQUENCES(5'-3') | |
| --- | --- | --- |
|  | Forward | Reverse |
| Sequence-1 | CTAATCCAGGAGCGTTGGGACTTTT | AGGGTTCCTGCCTAGACCTGACTCA |
| Sequence-2 | GGGTGACCAAGCCTTCAAGAATGCG | GCCTCAGCCCACCGCTGCTAAGAAT |
